# The Health Sector Response to Gender-Based Violence and Sexual Reproductive Health Programs in the Commonwealth and Selected African Countries: Protocol for a Mixed Methods Systematic Review and Meta-Analysis

**DOI:** 10.2196/67571

**Published:** 2025-09-18

**Authors:** Refilwe Nancy Phaswana-Mafuya, Edith Phalane, Nompumelelo Zungu, Alfred Musekiwa, Lebogang Ramalepe, Kayla Bagg, Peter Nyasulu, Olive Shisana

**Affiliations:** 1 South African Medical Research Council Pan African Centre for Epidemics Research Extramural Unit Faculty of Health Sciences University of Johannesburg Johannesburg South Africa; 2 Public Health, Societies and Belonging Human Sciences Research Council Pretoria South Africa; 3 School of Nursing and Public Health University of KwaZulu-Natal Durban South Africa; 4 School of Health Systems and Public Health Faculty of Health Sciences University of Pretoria Pretoria South Africa; 5 Vanderbijlpark Campus, COMPRES Research Entity University of North-West Vanderbijlpark South Africa; 6 Evidence Based Solutions Consulting, Pty Ltd Cape Town South Africa; 7 Division of Epidemiology and Biostatistics, Department of Global Health Faculty of Medicine and Health Sciences Stellenbosch University Cape Town South Africa; 8 Department of Psychiatry and Mental Health University of Cape Town Cape Town South Africa

**Keywords:** gender-based violence, sexual and reproductive health rights, health rights, violence against women and girls, violence against women, Commonwealth, African countries, violence, sexual health, reproductive health, women’s health, female health, social factor, social disparity, social inequality, social inequity, systematic review, review, systematic review protocol

## Abstract

**Background:**

The intertwining nature of gender-based violence (GBV) and violence perpetrated against women and girls (VAWG), as well as sexual and reproductive health rights (SRHR), underlines the urgent need for the health sector to enhance the coordination of services to improve health outcomes. Importantly, GBV and VAWG are intricately linked to a spectrum of SRHR challenges, ranging from unintended pregnancies to severe maternal, gynecological, and mental health outcomes. Cumulative GBV had a more significant effect on abortion risk than associated variables. Recognizing the interplay between GBV, VAWG, and SRHR highlights the necessity for a comprehensive health sector response. A systematic review of the health sector response to GBV, VAWG, and SRHR will be conducted to understand the extent and array of health facility–based coordinated responses to GBV, VAWG, and SRHR; lessons learned; and successes and challenges in the Commonwealth and selected African countries.

**Objective:**

We aim to understand the context of GBV, VAWG, and SRHR by conducting a comprehensive review of health sector responses in different national, cultural, and socioeconomic contexts, and we aim to share best practices, experiences, and lessons learned.

**Methods:**

A mixed methods systematic review will be conducted following the Preferred Reporting Items for Systematic Reviews and Meta-Analyses Protocol (PRISMA-P) guidelines. The population, intervention, comparison, and outcome framework will be applied to screen and select relevant sources guided by the inclusion and exclusion criteria. The review will include relevant research papers published in the last 15 years and conducted in the 24 Commonwealth and 7 selected African countries. Electronic databases to be searched will include PubMed, Google Scholar, Science Direct, EBSCOhost, Web of Science, Embase, PsycINFO, Cochrane, CINAHL, Index Medicus for the Eastern Mediterranean Region, and POPline.

**Results:**

Ethics approval will be waived as the study will use data in the public domain. The project has been commissioned by the Commonwealth Secretariat (2022-2025). The database search, data screening, and data extraction process for the review will be completed by September 2025. A manuscript will be submitted to a peer-reviewed international journal by November 2025. The initial online database searches, citations of eligible studies, and Microsoft Copilot identified 38,200 studies focusing on GBV, VAWG, and SRHR interventions. To date, 60 studies have been found eligible for inclusion in the review. The majority of these studies were conducted in eastern Africa (n=34), South Africa (n=14), and Asia (n=13). Evidence generated from this review will be made available through journal publications, seminars and workshops with key stakeholders, ministries of health, and local and international conferences.

**Conclusions:**

The study will generate evidence to inform recommendations on addressing and mitigating the effects of GBV and VAWG on SRHR outcomes and coordinated services in the health sectors of Commonwealth and selected African countries.

**Trial Registration:**

PROSPERO CRD42024520594; https://www.crd.york.ac.uk/PROSPERO/view/CRD42024520594

**International Registered Report Identifier (IRRID):**

PRR1-10.2196/67571

## Introduction

Gender-based violence (GBV) remains a critical public health issue, particularly affecting women, girls, and individuals with nonbinary gender identities in the Commonwealth and most African nations [1-4]. In a survey conducted by the World Health Organization (WHO), more than a quarter (27%) of women between the ages of 14 and 50 years reported having experienced physical or sexual violence, or both, from an intimate partner [1]. These individuals often face systemic social injustices as well as limited empowerment regarding GBV and sexual and reproductive health rights (SRHR) [4-8] and entrenched patriarchal norms that perpetuate violence and hinder access to essential health services [1,2]. GBV manifests both within and outside the home, with women and girls predominantly experiencing violence in domestic settings, while men often encounter it in public spaces. The forms of violence include physical, emotional, and sexual abuse, all of which adversely affect the survivor’s physical, social, and emotional well-being [1,2,9,10]. The consequences of GBV on SRHR are profound. Survivors may experience unintended pregnancies, pregnancy complications, and adverse maternal health outcomes such as low birth weight, miscarriage, and maternal mortality [11-14]. Additionally, GBV can lead to early sexual initiation, unprotected sex, and heightened susceptibility to sexually transmitted infections (STIs), particularly HIV [15-17]. Intimate partner violence also has a higher tendency to increase unwanted pregnancies and abortions. These experiences often occur in health care environments that are gender-insensitive, judgmental, discriminatory, and stigmatizing, lacking privacy and confidentiality, thereby deterring individuals from seeking SRHR services [15].

Furthermore, GBV can result in gynecological issues, such as genital and reproductive tract infections [11]. GBV is also implicated in mental health conditions such as depression, anxiety, and posttraumatic stress disorder [18-20]. These conditions impair individuals’ ability to make informed decisions about their SRHR. Factors like insufficient sexual empowerment, lack of emotional and social support, and inadequate mental health services compound the impact of GBV on SRHR outcomes. Studies have demonstrated that GBV is linked to induced and repeat abortions [21-23]. Notably, the cumulative effect of GBV victimization on the likelihood of abortion surpasses that of nonsexual childhood trauma and demographic factors [24]. Across various African countries, including Angola, Chad, Democratic Republic of the Congo, Gabon, Benin, Burkina Faso, Côte d’Ivoire, Gambia, Mali, Comoros, Rwanda, Uganda, Malawi, and Zambia [25], GBV has a stronger correlation with pregnancy termination among adolescent girls and young women. This underscores the need to view GBV-related induced abortion as a pressing public health issue. The impact of GBV extends to the use of reproductive health care services. Survivors may delay seeking care due to unwelcoming health care environments that lack privacy and confidentiality [13,14,24,26]. For instance, survivors of GBV may not have access to contraception; STI treatment or prevention services; HIV testing, prevention, and treatment services; or obstetric and gynecological care [13,27,28]. They may also be unable to access mental health services from professionals trained to work with GBV survivors, who are equipped to offer a safe, supportive, and nonjudgmental environment to discuss their experiences and address any associated psychological and emotional effects of GBV [19,20,29].

Addressing the intersection of GBV, violence against women and girls (VAWG), SRHR, and the health sector necessitates a coordinated and comprehensive response. The health sector must offer survivor-centered care that addresses the physical, mental, and social consequences of GBV. Collaboration with other sectors is essential to tackle the root causes of violence and prevent its recurrence. Providing these services enables survivors to regain control over their reproductive health and make informed decisions about abortion, their bodies, and their futures [13,14,30].

Target 3.7 of the United Nations Sustainable Development Goals (SDGs) and the Minimum Initial Service Package emphasize the critical importance of SRHR in health and development. However, various barriers and SRHR risks persist for women and girls, including individuals of all gender identities and sexual orientations, in accessing SRHR services. These barriers range from poor sanitation, inadequate resources and equipment, and substandard health facility conditions to the stigma surrounding GBV and SRHR services, such as judgmental attitudes, discrimination, and labeling, which prevent survivors from accessing and using SRHR services [31]. Health sector responses to GBV are inadequate in most Commonwealth and African countries [30]. This is mainly because of the inability to implement practical interventional strategies. These challenging matters are further compounded by the demand for more capacity in many health care systems to coordinate a multisector response. The Commonwealth Secretariat, health ministries in African countries, and stakeholders are well equipped to support developing a coordinated health sector response. A multisector, coordinated response may significantly reduce rates of GBV and SRHR-related issues. This systematic review aims to consolidate evidence on the health sector’s response to GBV, VAWG, and SRHR, focusing on the burden and diversity of health facility–based coordinated responses in the Commonwealth and selected African countries.

## Methods

### Study Design

A mixed methods systematic review will be carried out following the Preferred Reporting Items for Systematic Reviews and Meta-Analyses Protocol (PRISMA-P) guidelines [32] (Multimedia Appendix 1) and the Enhancing Transparency in Reporting the Synthesis of Qualitative Research recommendations [33]. The mixed methods approach will incorporate both qualitative and quantitative analyses of the studies selected for the review.

### Ethical Considerations

This systematic review will use data that are available in the public domain, so it is exempt from ethics approval. We will disseminate the findings of this systematic review on multiple platforms, including, among others, international peer-reviewed journals, webinars, stakeholder engagement sessions, digital platforms, and conferences. This systematic review protocol was registered with the International Prospective Register for Systematic Reviews (PROSPERO) with the registration number CRD 42024520594.

### Inclusion and Exclusion Criteria

The population, intervention, comparison, and outcome (PICO) framework will inform the criteria for including and excluding papers during the screening and for selecting relevant resources [34]. The review will include published literature from the last 15 years. Studies of any design focused on health sector responses to GBV, VAWG, and SRHR in the Commonwealth and selected African countries will be included. Documents beyond 15 years, commentaries, and documents not available as full texts after a request from the corresponding authors, as well as those not conducted in the 24 Commonwealth and the 4 selected African countries outside the Commonwealth nations, will be excluded from the review (Textbox 1).

**Inclusion and exclusion criteria using the population, intervention, comparison, outcome framework.**

**Inclusion criteria**

- Population: studies focusing on health sector responses to gender-based violence (GBV), violence perpetrated against women and girls (VAWG), and sexual and reproductive health rights (SRHR) in the Commonwealth and selected African countries among women, men, girls, and boys published in the last 15 years (2011-2025) and reported in English
- Intervention: studies that implemented interventions or strategies to prevent and control GBV and VAWG as well as mitigate negative SRHR outcomes
- Comparison: studies that compare different types of GBV, VAWG, and SRHR interventions
- Outcomes: studies that report as main outcomes VAWG, GBV, and SRHR knowledge, attitudes, confidence, and readiness; health-related behavior and practices (eg, identification and disclosure of VAWG and GBV; and provision and uptake of referrals and VAWG, GBV, and SRHR services

**Exclusion criteria**

- Studies not reporting on at least one aspect of the health sector response to GBV, VAWG, and SRHR in the Commonwealth and selected African countries
- Studies published prior to 15 years
- Studies not written in English

### Other Key Factors for Inclusion

We include studies reporting on GBV, VAWG, and SRHR interventions in the 31 Commonwealth and selected African countries. These countries include Malta, Fiji, the United Kingdom, New Zealand, Grenada, Canada, Guyana, Barbados, Antigua and Barbuda, Bangladesh, India, Malaysia, Pakistan, Sri Lanka, Burkina Faso, Central African Republic, Ghana, Guinea Bissau, Kenya, Sierra Leone, South Africa, Ethiopia, Congo, Tanzania, Rwanda, Benin, Senegal, Namibia, and Malawi. Depending on the country’s context, GBV, VAWG, and SRHR services can be rendered at any level of the health care system. Relevant peer-reviewed studies published in reputable journals available as full texts will be considered for inclusion.

### Search Strategy and Databases

A comprehensive search strategy will be developed to identify relevant studies. The search strategy for the systematic review will be guided by the PICO framework and Medical Subject Heading terminology (Multimedia Appendix 2) [35]. The process of developing the search strategy will involve searching multiple databases. Data sources to be used will include PubMed, Google, Google Scholar, Science Direct, EBSCOhost, Web of Science, Medline, Embase, PsycINFO, Cochrane, CINAHL, Index Medicus for the Eastern Mediterranean Region, POPLine, Lilacs, the WHO Reproductive Health Library, and ClinicalTrials.gov. Abstracts and full-text articles will be reviewed using an online systematic review tool (Covidence) and a referencing tool (EndNote). Additionally, reference lists of prior systematic reviews on GBV, VAWG, and SRHR domains in the Commonwealth and selected African countries will be reviewed for potentially relevant studies. These multiple complementary search strategies will be used to ensure that the search is as exhaustive as possible. The purpose is to have a wide net to harness the diversity of GBV, VAWG, and SRHR literature.

### Quality Appraisal and Screening

Published literature will be retrieved from the online database search engines and imported into Covidence before the screening process. The quality appraisal of the studies will be done following the Critical Appraisal Skills Programme systematic review checklist, which examines the validity, precision, and generalizability of the research [23]. All duplicates will be excluded from the software during the importation phase. Two authors will screen the imported articles by title and abstract. The articles that meet the criteria based on their titles and abstracts will further be screened as full texts by the 2 reviewers. The screening processes will involve dividing the articles and reports into 3 groups: yes, no, and maybe. A third member will weigh in and make the final decision if there are different opinions.

### Data Extraction and Evidence Synthesis

A data extraction database will be in place to extract and populate the information. Information from the eligible articles will be extracted with a Microsoft Excel data extraction tool using relevant headings, such as the study and author names, year of publication, country, study design, study population, interventions, and key findings.

### Statistical Analysis

We will analyze data abstracted from included studies to assess the effect of the intervention applied in preventing and controlling GBV and VAWG. Statistical analysis will use Stata (version 18; StataCorp) and R (version 4.4; R Foundation for Statistical Computing). We will analyze dichotomous outcome data as odds ratios (ORs) and/or risk ratios (RRs). All continuous data will be analyzed as mean differences (MDs) with corresponding SDs. We will report all estimates with 95% CIs. We will conduct a meta-analysis if at least 2 similar studies are available so that we can determine the pooled OR, RR, or MD of each of the individual studies. We will present the results graphically as forest plots. We will quantitatively assess, using the *I*^2^ statistic, any heterogeneity that is identified in the different study designs or participants’ characteristics that could lead to differences in outcomes between these studies. Where no significant heterogeneity (*I*^2^<50%) exists, fixed effects models will be used to pool the results of the studies. Random effects models will be used if there is either observable or significant heterogeneity (*I*^2^≥50%). Where appropriate, we will use a fixed effects model to obtain pooled estimates of GBV and related propagating factors.

We will perform subgroup analysis to investigate the differences between GBV, VAWG, and SRHR interventions and outcomes among different countries within southern, central, western, and eastern sub-Saharan Africa. This will be done should there be sufficient data to draw upon for such a comparative analysis. Should the meta-analysis include more than 10 papers, a funnel plot will be generated so that publication bias can be evaluated. Statistical tests, if appropriate, will be done for funnel plot asymmetry (Egger test, Begg test, or Harbord test). We will use a meta-regression to assess factors associated with GBV and related outcomes. Lastly, we will conduct a leave-one-out sensitivity analysis for studies that are found to be outliers to evaluate the robustness of the pooled estimates. For qualitative data amenable to meta-synthesis, textual data will be aggregated using the JBI’s Qualitative Assessment and Review Instrument and narrative tools. The data will be analyzed using framework methodologies, using both case-based and theme-based approaches.

### Integration of the Qualitative and Quantitative Data

Triangulation, as well as complementary and joint display, will be used to integrate the quantitative and qualitative data during the analysis phase. The triangulation method is used to harness data from different sources to explain a particular phenomenon, with the purpose of finding convergence or divergence in the results [36]. Additionally, the complementary method will be used, wherein qualitative data will be used to explain observations seen in the quantitative data or vice versa [37]. For example, the qualitative responses can be used to explain why certain GBV or VAWG interventions were adequate or not in preventing and controlling GBV and VAWG. Additionally, this method can be used to assess how an intervention mitigates the effects of GBV and VAWG on SRHR outcomes. Joint displays such as side-by-side comparisons will be used to integrate GBV and VAWG intervention facilitators and barriers with quantitative data (ie, a meta-analysis) to show the effectiveness of a GBV or VAWG intervention [38,39].

### Assessing the Certainty of Evidence Using Grading of Recommendations Assessment, Development and Evaluation

We will use the Grading of Recommendations Assessment, Development and Evaluation (GRADE) to assess the certainty of evidence. This approach is important for evaluating the overall quality or certainty of the evidence on the key outcomes provided by the individual studies included in the review. In this review, as stated earlier, 2 reviewers, at a minimum, will assess the certainty of evidence (by assessing the quality of evidence or the confidence limits in the effect estimates). This will cover the following key domains: imprecision, inconsistency, risk of bias, indirectness, and publication bias. The certainty of evidence will be graded as high, moderate, low, or very low. For each of the outcomes, we will present an overall rating of the certainty of the evidence in the form of a table summarizing the findings. This will be presented with the number of studies, participants, study types, and each outcome’s relative and absolute effects. We will summarize publication bias using funnel plots and the Egger regression test technique.

### Search Results and Study Selection

Published studies were screened and selected by 2 independent reviewers. These was afterwards retrieved and uploaded into Endnote (version X9). Duplicates were screened and removed from further processing. The titles and abstracts of the studies were screened to remove all nonqualifying and irrelevant studies. The full-text articles meeting the inclusion criteria were screened and added to the selected list. Any study that did not qualify for inclusion at this stage was excluded based on having a wrong outcome, study design, or population. Any disagreements between the reviewers regarding study selection were resolved by a fourth reviewer, who intervened and resolved the disagreement.

## Results

**Figure 1:**
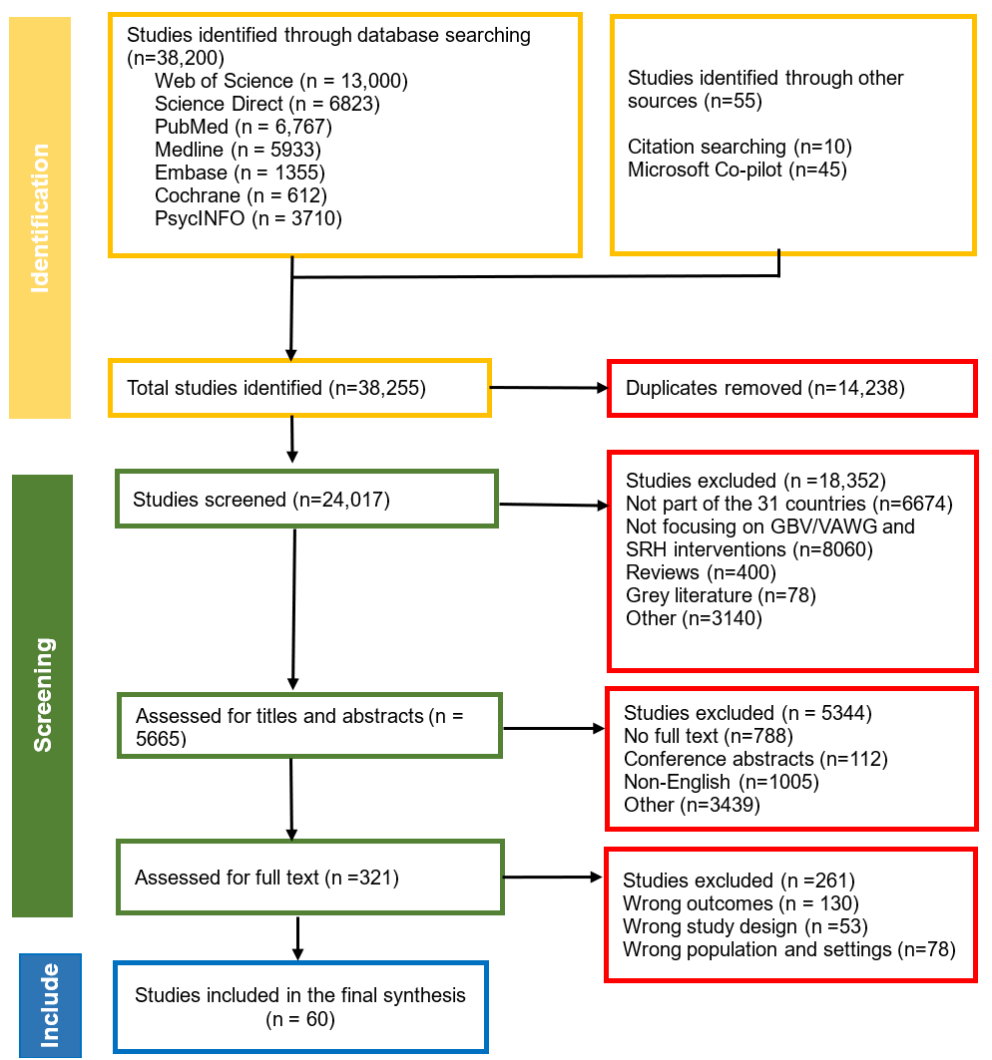
Preferred Reporting Items for Systematic Reviews and Meta-Analyses Protocol (PRISMA-P) flow chart summarizing the screening process for including eligible articles as of July 21, 2025. GBV: gender-based violence; SRHR: sexual reproductive health and rights; VAWG: violence against women and girls.

The PRISMA-P flow chart in Figure 1 summarizes the process of selecting the articles included in this review. A total of 38,200 studies were initially identified through online database searches, as follows: Web of Science (n=13,055), Science Direct (n=6823), PubMed (n=6767), Medline (n=5933), Embase (n=1355), Cochrane (n=612), and PsycINFO (n=3710). Additional studies were identified through reference lists from the eligible studies (n=10) and Microsoft Copilot (n=45). Duplicates were removed after the studies were added to RefWorks and Covidence; there remained 14,238 studies. A total of 24,017 studies were screened, and 18,352 were excluded due to reasons such as not focusing on the 31 Commonwealth and selected African countries (n=6674) and not focusing on GBV, VAWG, and SRHR (n=8060). After the title and abstract assessment, 5344 studies were removed due to there being no full text available (n=788), not being in English (n=1005), or being conference abstracts (n=122). The remaining 321 studies were assessed as full texts, and 60 studies were included in the final review. In terms of geographical distribution, most of the studies came from eastern Africa (n=34), South Africa (n=14), and Asia (n=13). The literature search, data screening, and data extraction for the review will be completed by September 2025. A completed manuscript will be ready by the end of November 2025 and will be submitted for peer review to an international journal.

## Discussion

### Overview

This systematic review will unravel the body of evidence available related to the burden of GBV and health facility–coordinated responses to GBV, VAWG, and SRHR challenges, as well as potential mitigating approaches. It will apply robust methods to address external and internal validity. The external validity assessment will use PICO to streamline and delineate the attributes of the populations, interventions, comparisons, and outcomes. Furthermore, internal validity will address the risk of bias in various domains. The evidence generated from this review will thus provide lessons learned over time, successes observed, and challenges to be mitigated in implementing effective responses to minimize GBV and related SRHR challenges in the Commonwealth and selected African countries. Furthermore, it is anticipated that this systematic review will contribute toward identifying knowledge gaps, strengthening continuity and scale-up of effective interventions, guiding the development of future research agendas, and contributing toward gender equality, inclusivity, and social justice, as well as making the case for future investments. The review will use the PRISMA-P guidelines to ensure the consistency of the methodology and to guide the collection of information and material. Including diverse sources (eg, peer-reviewed publications) across different databases will allow the selection of the most relevant studies, enhance cross-validation of data from different sources, and minimize gaps.

### Study Limitations

Studies on GBV and SRHR might not be reported adequately due to stigma related to the subject, which might result in publication bias. The review may include research from various settings with different health care systems and cultural norms, which may limit the generalizability of the findings. This might be due to geographic and socioeconomic disparities in how GBV is viewed and managed when reported. Comprehensive analysis may also be limited by incomplete reporting of relevant outcomes of GBV events.

### Implications of the Expected Review Findings

The strength of this review will be to pool data from other studies by searching comprehensive databases and describing SRHR interventions that have been implemented to prevent and control GBV. The findings should help develop or modify policies on GBV and related challenges, clinical practice, and research in the Commonwealth and selected African countries. This review will draw attention to the need for more pertinent, focused programs to protect women and girls as well as for effective health care services to support women and girls who have experienced GBV. The results from this review could also help highlight differences in health outcomes according to risk profiles such as low socioeconomic status and geographical characteristics, reinforcing the need for equitable resource allocation and focused public health interventions for GBV among affected individuals in resource-limited settings. These findings may also shape health policies that support better access to care and treatment among GBV survivors, as well as reinforce SRHR and active GBV surveillance and education and discourage practices that may increase GBV against women and girls. This review may also point out gaps in the current body of knowledge about GBV and SRHR and associated impediments to effective reporting, care, and psychosocial counselling services. We believe that the evidence generated from this review will contribute to achieving SDG 3.3 and lead to improved interventions to eliminate GBV and enhance SRHR to improve women’s health.

## Appendix

Multimedia Appendix 1 PRISMA-P checklist.

Multimedia Appendix 2 Sample search strategy in PubMed.

## Abbreviations

GBV: gender-based violence
GRADE: Grading of Recommendations Assessment, Development and Evaluation
MD: mean difference
OR: odds ratio
PICO: population, intervention, comparison, outcome
PRISMA: Preferred Reporting Items for Systematic Reviews and Meta-Analyses-Protocol
PROSPERO: International Prospective Register of Systematic Reviews
RR: risk ratio
SDG: Sustainable Development Goals
SRHR: sexual and reproductive health rights
STI: sexually transmitted infection
VAWG: violence against women and girls
WHO: World Health Organization

## Data Availability

All data generated or analyzed during this study will be included in the published article and its supplementary information files.

## References

[1] (2024). Violence against women. World Health Organization.

[2] Facts and figures: Ending violence against women. UN Women.

[3] (2021). Measuring the prevalence of online violence against women. The Economist Intelligent Unit.

[4] Perrin N, Marsh M, Clough A, Desgroppes A, Yope Phanuel C, Abdi A, Kaburu F, Heitmann S, Yamashina M, Ross B, Read-Hamilton S, Turner R, Heise L, Glass N (2019). Social norms and beliefs about gender based violence scale: a measure for use with gender based violence prevention programs in low-resource and humanitarian settings. Confl Health.

[5] Blondeel K, de Vasconcelos S, García-Moreno Claudia, Stephenson R, Temmerman M, Toskin I (2018). Violence motivated by perception of sexual orientation and gender identity: a systematic review. Bull World Health Organ.

[6] Coates A, Allotey P (2023). Global health, sexual and reproductive health and rights, and gender: square pegs, round holes. BMJ Glob Health.

[7] Wigle JM, Paul S, Birn A, Gladstone B, Kalolo M, Banda L, Braitstein P (2022). Participation of young women in sexual and reproductive health decision-making in Malawi: Local realities versus global rhetoric. PLOS Glob Public Health.

[8] Durojaye E, Mirugi-Mukundi G, Ngwena C (2021). Advancing Sexual and Reproductive Health and Rights in Africa: Constraints and Opportunities.

[9] Dicola D, Spaar E (2016). Intimate partner violence. Am Fam Physician.

[10] Forms of violence. European Institute for Gender Equality.

[11] Grose RG, Chen JS, Roof KA, Rachel S, Yount KM (2021). Sexual and reproductive health outcomes of violence against women and girls in lower-income countries: a review of reviews. J Sex Res.

[12] Bagade T, Chojenta C, Harris M, Oldmeadow C, Loxton D (2022). The human right to safely give birth: data from 193 countries show that gender equality does affect maternal mortality. BMC Pregnancy Childbirth.

[13] Lewis NV, Munas M, Colombini M, d'Oliveira AF, Pereira S, Shrestha S, Rajapakse T, Shaheen A, Rishal P, Alkaiyat A, Richards A, Garcia-Moreno CM, Feder GS, Bacchus LJ (2022). Interventions in sexual and reproductive health services addressing violence against women in low-income and middle-income countries: a mixed-methods systematic review. BMJ Open.

[14] Bolarinwa OA, Boikhutso T (2022). A mixed-method analysis of inequalities associated with adverse sexual and reproductive health outcomes and the requisite interventions among young women in Durban informal settlements, South Africa. Front Public Health.

[15] Mukanangana F, Moyo S, Zvoushe A, Rusinga O (2014). Gender based violence and its effects on women's reproductive health: the case of Hatcliffe, Harare, Zimbabwe. Afr J Reprod Health.

[16] Geller RJ, Decker MR, Adedimeji AA, Weber KM, Kassaye S, Taylor TN, Cohen J, Adimora AA, Haddad LB, Fischl M, Cunningham S, Golub ET (2020). A prospective study of exposure to gender-based violence and risk of sexually transmitted infection acquisition in the Women's Interagency HIV Study, 1995-2018. J Womens Health (Larchmt).

[17] Tazinya RMA, El-Mowafi IM, Hajjar JM, Yaya S (2023). Sexual and reproductive health and rights in humanitarian settings: a matter of life and death. Reprod Health.

[18] Sexual and Reproductive Health and Research. World Health Organization.

[19] Gelaye B, Arnold D, Williams MA, Goshu M, Berhane Y (2009). Depressive symptoms among female college students experiencing gender-based violence in Awassa, Ethiopia. J Interpers Violence.

[20] Patel R, Gupte SS, Srivastava S, Kumar P, Chauhan S, Govindu MD, Dhillon P (2021). Experience of gender-based violence and its effect on depressive symptoms among Indian adolescent girls: evidence from UDAYA survey. PLoS One.

[21] Nguyen Phuong Hong, Nguyen Son Van, Nguyen Manh Quang, Nguyen Nam Truong, Keithly S, Mai Lan Tran, Luong Loan Thi Thu, Pham Hoa Quynh (2012). The association and a potential pathway between gender-based violence and induced abortion in Thai Nguyen province, Vietnam. Glob Health Action.

[22] Pallitto CC, García-Moreno Claudia, Jansen HA, Heise L, Ellsberg M, Watts C, WHO Multi-Country Study on Women's Health and Domestic Violence (2013). Intimate partner violence, abortion, and unintended pregnancy: results from the WHO Multi-country Study on Women's Health and Domestic Violence. Int J Gynaecol Obstet.

[23] McCloskey L (2016). The effects of gender-based violence on women's unwanted pregnancy and abortion. Yale J Biol Med.

[24] Bohren MA, Vazquez Corona M, Odiase OJ, Wilson AN, Sudhinaraset M, Diamond-Smith N, Berryman J, Tunçalp Özge, Afulani PA (2022). Strategies to reduce stigma and discrimination in sexual and reproductive healthcare settings: a mixed-methods systematic review. PLOS Glob Public Health.

[25] Ahinkorah BO (2021). Intimate partner violence against adolescent girls and young women and its association with miscarriages, stillbirths and induced abortions in sub-Saharan Africa: Evidence from demographic and health surveys. SSM Popul Health.

[26] Baigry MI, Ray R, Lindsay D, Kelly-Hanku A, Redman-MacLaren M (2023). Barriers and enablers to young people accessing sexual and reproductive health services in Pacific island countries and territories: A scoping review. PLoS One.

[27] Akazili J, Kanmiki EW, Anaseba D, Govender V, Danhoundo G, Koduah A (2020). Challenges and facilitators to the provision of sexual, reproductive health and rights services in Ghana. Sex Reprod Health Matters.

[28] Huang K, Kumar M, Cheng S, Urcuyo AE, Macharia P (2022). Applying technology to promote sexual and reproductive health and prevent gender based violence for adolescents in low and middle-income countries: digital health strategies synthesis from an umbrella review. BMC Health Serv Res.

[29] Chomba E, Murray L, Kautzman M, Haworth A, Kasese-Bota M, Kankasa C, Mwansa K, Amaya M, Thea D, Semrau K (2010). Integration of services for victims of child sexual abuse at the university teaching hospital one-stop centre. J Trop Med.

[30] Norbu N, Zam K (2021). Assessment of health‐sector response to gender‐based violence at different levels of health facilities in Bhutan (2015–2016). World Med Health Policy.

[31] Ivanova O, Rai M, Kemigisha E (2018). A systematic review of sexual and reproductive health knowledge, experiences and access to services among refugee, migrant and displaced girls and young women in Africa. Int J Environ Res Public Health.

[32] Page M, McKenzie J, Bossuyt P, Boutron I, Hoffmann T, Mulrow C, Shamseer Larissa, Tetzlaff Jennifer M, Akl Elie A, Brennan Sue E, Chou Roger, Glanville Julie, Grimshaw Jeremy M, Hróbjartsson Asbjørn, Lalu Manoj M, Li Tianjing, Loder Elizabeth W, Mayo-Wilson Evan, McDonald Steve, McGuinness Luke A, Stewart Lesley A, Thomas James, Tricco Andrea C, Welch Vivian A, Whiting Penny, Moher David (2021). The PRISMA 2020 statement: an updated guideline for reporting systematic reviews. Syst Rev.

[33] Tong A, Flemming K, McInnes E, Oliver S, Craig J (2012). Enhancing transparency in reporting the synthesis of qualitative research: ENTREQ. BMC Med Res Methodol.

[34] Helfer B, Samara M, Leucht S (2015). Experiences with Covidence in preparing a comprehensive systematic review. Cochrane Collaboration.

[35] (2022). CASP Checklists. Critical Appraisal Skill Programme.

[36] Arias Valencia María Mercedes (2022). Principles, scope, and limitations of the methodological triangulation. Invest Educ Enferm.

[37] Lochmiller CR (2018). Complementary Research Methods for Educational Leadership and Policy Studies.

[38] Guetterman Timothy C, Fetters Michael D, Creswell John W (2015). Integrating quantitative and qualitative results in health science mixed methods research through joint displays. Ann Fam Med.

[39] Bradt Joke, Potvin Noah, Kesslick Amy, Shim Minjung, Radl Donna, Schriver Emily, Gracely Edward J, Komarnicky-Kocher Lydia T (2015). The impact of music therapy versus music medicine on psychological outcomes and pain in cancer patients: a mixed methods study. Support Care Cancer.

