# Supplementary material for: The Health Sector Response to Gender-Based Violence and Sexual Reproductive Health Programs in the Commonwealth and Selected African Countries: Protocol for a Mixed Methods Systematic Review and Meta-Analysis
Source: JMIR Res Protoc. 2025 Sep 18;14:e67571. doi: 10.2196/67571 (PMC12491884; doi:10.2196/67571)
Supplement: Multimedia Appendix 2 [file resprot_v14i1e67571_app2.docx]

**Multimedia Appendix B – Sample search strategy in PubMed**

| **Search** | **Query** |
| --- | --- |
| #1 | **Search: (Health system response)**  “Health system response” OR “health response” “interventions” OR “programmes” OR “strategies” OR “health promotion” OR “model of care” OR “models of service” OR “programme development” OR “programme evaluation” OR “prevention” OR “capacity building” OR “guidelines and protocols” OR referral system” |
| #2 | **Search: (Gender-based violence OR GBV OR intimate Partner Violence OR Abuse OR Rape OR)**  "Gender-based violence" OR "Gender-based violence" OR "GBV" OR “Violence” OR "intimate partner violence" OR “IPV” OR “domestic violence” OR "Rape" OR “Abuse" OR "Sexual abuse" OR "physical abuse" OR "violence against women and girls" OR "VAWG" OR "wife-beating" OR partner violence" OR "relationship violence" OR "couple violence" OR relationship aggression" OR "marital abuse" OR "marital violence" OR "spousal violence" OR "spousal abuse" OR “sexual health” OR “sex offenses” |
| #3 | **Search: (sexual and reproductive health OR SRH OR sexual health OR reproductive health OR Sexual Reproductive Health Rights OR SRHR)**  "sexual and reproductive health" OR "sexual health" OR "reproductive health" OR "SRH" OR ‘sexual reproductive health rights’ OR “sexual reproductive health rights” “SRHR” “general sexual and reproductive health” OR “pregnancy OR family planning” OR “contraception” OR “abortion” OR “pregnancy termination” OR “postabortion” OR "post-abortion" OR "post abortion" OR “feticide” OR “foeticide” OR “prenatal healthcare” OR “antenatal health care” OR “HIV” OR “AIDS” “HIV/AIDS” OR “STIs” OR “prevention of mother-to-child transmission” OR “PMTCT” OR “maternal and newborn health” |
| # 4 | **Search: (commonwealth countries OR commonwealth states OR selected African countries)**  "Commonwealth countries"[MeSH Terms] OR ("commonwealth states"[All Fields] AND “Africa” OR “African countries” AND "South Africa"[All Fields] OR "Ghana"[All Fields] OR "Namibia"[All Fields] OR "Burkina Faso"[All Fields])  Burkina Faso, Central African Republic, Ghana, Guinea Bissau, Kenya, Sierra Leone, South Africa (partial), United Republic of Tanzania, Senegal, Malawi, Rwanda, Benin, Ethiopia, Democratic Republic of Congo, Namibia, Bangladesh, India, Malaysia, Pakistan, Sri Lanka, Caribbean and Americas, Antigua and Barbuda, Barbados, Canada, Grenada, Guyana, Europe, Republic of Cyprus, Malta, UK of Great Britain and Northern Ireland, Australia, New Zealand |
| # 5 | # 1 AND # 2 AND # 3 AND # 4 AND #5 |
